# Leukemogenic rearrangements at the mixed lineage leukemia gene (*MLL*)—multiple rather than a single mechanism

**DOI:** 10.3389/fcell.2015.00041

**Published:** 2015-06-25

**Authors:** Boris Gole, Lisa Wiesmüller

**Affiliations:** Division of Gynecological Oncology, Department of Obstetrics and Gynecology, Ulm UniversityUlm, Germany

**Keywords:** *MLL* breakpoint cluster region, infant acute leukemia, therapy-related leukemia, apoptosis, replication stress, error-prone DNA repair

## Abstract

Despite manifold efforts to achieve reduced-intensity and -toxicity regimens, secondary leukemia has remained the most severe side effect of chemotherapeutic cancer treatment. Rearrangements involving a short telomeric <1 kb region of the mixed lineage leukemia (*MLL*) gene are the most frequently observed molecular changes in secondary as well as infant acute leukemia. Due to the mode-of-action of epipodophyllotoxins and anthracyclines, which have widely been used in cancer therapy, and support from *in vitro* experiments, cleavage of this *MLL* breakpoint cluster hotspot by poisoned topoisomerase II was proposed to trigger the molecular events leading to malignant transformation. Later on, clinical patient data and cell-based studies addressing a wider spectrum of stimuli identified cellular stress signaling pathways, which create secondary DNA structures, provide chromatin accessibility, and activate nucleases other than topoisomerase II at the *MLL*. The *MLL* destabilizing signaling pathways under discussion, namely early apoptotic DNA fragmentation, transcription stalling, and replication stalling, may all act in concert upon infection-, transplantation-, or therapy-induced cell cycle entry of hematopoietic stem and progenitor cells (HSPCs), to permit misguided cleavage and error-prone DNA repair in the cell-of-leukemia-origin.

## Introduction

The *KMT2A* gene, better known as *MLL* (mixed lineage or myeloid/lymphoid leukemia) encodes a lysine (K)-specific histone methyltransferase 2A, which functions as an epigenetic regulator of transcription (Daniel and Nussenzweig, 2012; Takeda et al., 2013). The enzyme is primarily connected to hematopoietic and embryonic development (Hess et al., 1997), but was also described to contribute to the S-phase DNA damage checkpoint (Liu et al., 2010). The gene spans the breakpoint cluster region at chromosomal position 11q23, frequently rearranged in acute leukemia, especially in therapy-related and infant cases (Ziemin-van der Poel et al., 1991; Emerenciano et al., 2007; Cowell and Austin, 2012). Despite over two decades of efforts, the reasons underlying the exceptionally high breakability of the *KMT2A/MLL* locus are still not clear. The same is true also for the mechanisms leading to its rearrangements. In this review we summarize the facts and hypotheses which in our view are relevant for understanding of *KMT2A/MLL* breakage and rearrangements, with a focus on therapy-related cases.

## Incidence and risk of *MLL* rearrangements

Rearrangements of the *MLL* gene were found in 5.2% of all the acute myeloid leukemia (AML) cases and in 22% of all the acute lymphoid leukemia (ALL) cases (De Braekeleer et al., 2005). Patients with *MLL* rearrangements have poorer prognosis than the ones without, with shorter event free and overall survival rates (Tamai et al., 2008; Cerveira et al., 2012; Chen et al., 2013). Interestingly, myelodsyplastic syndrome (MDS) patients with the 11q23 rearrangement *t*_(2; 11)(*p*21; *q*23)_ which does not affect the *MLL* gene but rather upregulates a downstream lying miRNA *MIR125B1* resulting in inhibited primary human CD34^+^ cell differentiation, have a favorable prognosis (Bousquet et al., 2008; Dvorak et al., 2014).

In childhood ALL, *MLL* rearrangements are found in 44–85% of the infants (<1 year old), which decreases down to 3% in the elder (1–10 years) patients (Emerenciano et al., 2007, 2013; Al-Sudairy et al., 2014). Data from siblings revealed that both genetic and environmental risk factors are implied in childhood ALL (Schmiegelow et al., 2012). As *MLL* rearrangements in these patients can arise during *in utero* fetal hematopoiesis (Gale et al., 1997), prenatal exposure and consequently lifestyle of the mother are highly relevant for development of this type of leukemia. Increased *MLL* rearrangements were indeed observed in amniocytes from long-term smokers in a small prospective study (de la Chica et al., 2011), as well as a statistically significant association between intake of hormones during pregnancy and risk of *in utero MLL* rearrangements in a study enrolling several 100 children (Pombo-de-Oliveira et al., 2006). Estrogen, in particular, was demonstrated to induce *MLL* breakage and rearrangements in cultured lymphoblastoid cells (Schnyder et al., 2009). The most prominent hints to exogenous *MLL* destabilizing sources stem from reports on the intake of dietary flavonoids during pregnancy. Bioflavonoids, such as quercetin, hydroquinone or genistein which are present in citrus, certain types of berries and root vegetables, can induce *MLL* cleavage and rearrangements *ex vivo* in human hematopoietic stem and progenitor cells (HSPCs) isolated from umbilical cord blood (Strick et al., 2000; van Waalwijk van Doorn-Khosrovani et al., 2007) and *in utero* in mice (Vanhees et al., 2011).

With rearrangements found in ~40% of therapy-related acute leukemia/myelodsyplastic syndrome (t-AL/MDS), *MLL* is the most frequently rearranged gene in t-AL (Shivakumar et al., 2008; Pullarkat et al., 2009; Shim et al., 2010; Abdulwahab et al., 2012; Cowell and Austin, 2012). The lifelong risk of t-AL/MDS in patients receiving chemo- and/or radio-therapy was found to be 0.2% among US cancer patients (Morton et al., 2013). Depending on the type of primary cancer and especially the therapy used, it varied between 0.02 and 12% (Maddams et al., 2011; Abdulwahab et al., 2012; Ezoe, 2012; Koontz et al., 2013; Morton et al., 2013). The majority of cases occur during the first 5 years after treatment of primary cancer (Shivakumar et al., 2008; Pullarkat et al., 2009; Ezoe, 2012; Koontz et al., 2013). Though causalities are often difficult to assess as patients receive complex treatments, the highest (up to 12%) incidence of t-AL was observed in patients treated with topoisomerase II inhibitors such as epipodophyllotoxins and anthracyclines (Abdulwahab et al., 2012; Ezoe, 2012). Use of topoisomerase II inhibitors also seemed connected to over 90% of all the 11q23 rearrangements in patients receiving chemotherapy (Shivakumar et al., 2008). A causal link between topoisomerase II inhibitor treatment and *MLL* rearrangements was supported by *in vitro* data on stable *MLL* rearrangements in human embryonic and hematopoietic stem cells (HSCs) treated with topoisomerase II inhibitor etoposide (Libura et al., 2008; Bueno et al., 2009).

Interestingly, bioflavonoids were connected to *MLL* rearrangements in infant ALL and were shown to inhibit human topoisomerase II (Strick et al., 2000), so that causes for infant ALL might be similar to the ones for t-AL. Both types of leukemia are characterized by short latency after the initiation event- exposure of the hematopoietic system to the *MLL* destabilizing agents *in utero* or during primary cancer treatment, respectively. To the best of our knowledge no links between bioflavonoids/topoisomerase II-inhibiting compounds and *MLL* rearrangements in *de novo* AL were described so far. Striking similarities between infant and therapy-related AL were, however, found when comparing the breakage distribution within the *MLL* as discussed below.

## Distribution of break sites leading to leukemic rearrangements

**Figure 1:**
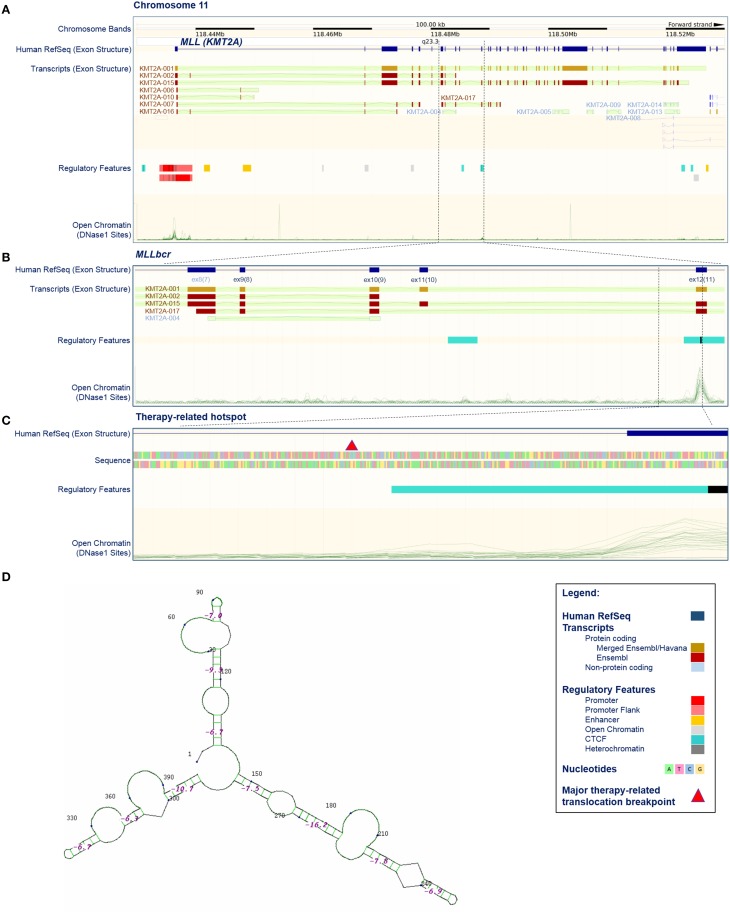
**Position, structure, and regulatory features of** ***MLL*** **breakpoint cluster region. (A–C)** Selected features of the *KMT2A/MLL* gene based on current issue of Ensembl data (release 79, March 2015; http://www.ensembl.org/index.html; Cunningham et al., 2015). **(A**) *KMT2A/MLL* position on chromosome 11, exon structure according to the current reference sequence (Human RefSeq), transcripts (protein coding and non-protein coding), regulatory features and open chromatin (DNase I sensitive) sites. Some transcripts harbor exons that are not acknowledged by the reference sequence (for example compare Human RefSeq, KMT2A-001 and KMT2A-002). Open chromatin sites are detectable in promoter-exon 1, exon 12 and exon 37. The position of the breakpoint cluster region (*MLLbcr*) is marked by dashed lines. **(B)** Enhanced view of the *MLLbcr* with exon structure of the reference sequence and transcripts, regulatory features and open chromatin (DNase I) sites. The position of the therapy-related hotspot is marked by dashed lines. **(C)** Enhanced view of a therapy-related hotspot flanking a major therapy-related translocation breakpoint described by Mirault et al. (2006), with exon structure, sequence, regulatory features and open chromatin (DNase I) sites. The major therapy-related breakpoint cluster (marked by red triangle) lies at the 5′ end of intron 11, just 3′ of the DNase I and CTCF-binding site found in 17/18 and 18/18 cell lines used in Ensembl, respectively. The indicated 399 bp segment was used in reporter studies with exogenous *MLLbcr* fragment (Boehden et al., 2004; Gole et al., 2014; Ireno et al., 2014). **(D)** Secondary structure of the 399 bp therapy-related hotspot. The structure was calculated using programs performing searches based on sequence, namely Quadruplex forming G-Rich Sequences (QGRS) Mapper (http://bioinformatics.ramapo.edu/QGRS/index.php) and M-Fold for hairpins (http://mfold.rna.albany.edu/?q=mfold).

More than 95% of the *MLL* rearrangements fall within a ~7.3 kb breakpoint cluster region (*MLLbcr*). This was originally described to range from exon 9 to intron 11/exon 12 of the *MLL* gene (Reichel et al., 2001; Meyer et al., 2013). However, according to the current reference sequence (GRCh38.p2, http://www.ncbi.nlm.nih.gov/projects/genome/assembly/grc/human/) this region corresponds to exon 8—intron 10/exon 11 (Figures 1A,B). For the purpose of clarity and comparability to publications cited in this review, we use the original numbering throughout this paper. Within the *MLLbcr* the distribution of breaks is not uniform, but rather forms two clusters which together harbor ~80% of all the breaks (Meyer et al., 2013). The centromeric cluster can be attributed mostly to cases of *de novo* AL in adults, while breaks from t-AL and infant ALL cluster at the telomeric part of the *MLLbcr* (Broeker et al., 1996; Cimino et al., 1997; Reichel et al., 2001; Cowell and Austin, 2012; Meyer et al., 2013), in particular, within a short ~600 bp translocation hotspot at the intron 11/exon 12 boundary (Mirault et al., 2006). Similar clustering to intron 11/exon 12 boundary can be found also when looking at breaks and rearrangements following topoisomerase II inhibitor and flavonoid treatments (van Waalwijk van Doorn-Khosrovani et al., 2007; Le et al., 2009), providing further support for a mechanistic connection between secondary and infant ALL.

Over 100 different rearrangements and over 60 translocation partners of *MLL* have been described so far, however most of them were reported only sporadically (Meyer et al., 2006, 2009). A handful of translocation partners, namely *AFF1/AF4, MLLT3/AF9, MLLT1/ENL, MLLT10/AF10*, and *MLLT4/AF6*, were found to be involved in 70–95% of the *MLL* translocations, with some differences in frequencies of individual partners in ALL vs. AML, infant vs. adult, and *de novo* vs. therapy-induced leukemia (Meyer et al., 2009, 2013; De Braekeleer et al., 2011; Cerveira et al., 2012; Emerenciano et al., 2013). Relatively narrow assortment of recurrent translocation partners most likely reflects some growth advantage of the mutant cells due to the particular rearrangement (Strick et al., 2006; Bueno et al., 2009). In line with this concept, examination of the blood samples from breast cancer and lymphoma patients undergoing topoisomerase II inhibitor treatment showed that *MLL* translocations are much more common than the actual t-AL cases (Le et al., 2009). Stable *MLL* rearrangements in human HSCs treated with etoposide did indeed increase the proliferative capacity of the cells (Libura et al., 2008; Bueno et al., 2009), though additional abnormalities seem to be needed for the completion of malignant transformation (Bueno et al., 2009). As a prominent example, the *MLL-AF9* fusion gene was shown to be actively transcribed (Betti et al., 2003) and the resulting fusion protein promoted expression of Hoxa9 transcription factor which has been associated with leukemic transformation (Muntean et al., 2010). Despite the fact that reciprocal gene fusions represent the majority of stable *MLL* rearrangements, a plethora of additional *MLL* aberrations namely gene-internal duplications, inversions, deletions, insertions, and complex rearrangements were identified in leukemia patients (Meyer et al., 2013), underscoring the fact that the *MLLbcr* bears intrinsic features causing exceptionally high fragility.

## Which enzymes cause *MLL* breakage?

Already in the 1990s, *MLLbcr* breakage and rearrangements causing secondary leukemia in patients undergoing chemotherapy were linked to the action of topoisomerase II inhibitors. Thus, cleavage by topoisomerase II at consensus sequences within a centromeric *MLLbcr* fragment was demonstrated *in vitro* (Felix et al., 1995). Translocation breakpoint junctions of a *t*_(4; 11)_ and a *t*_(9; 11)_ secondary leukemia patients were found to coincide with topoisomerase II sensitive sites, even though not consensus sequences, in the centromeric and telomeric *MLLbcr* fragment, respectively (Lovett et al., 2001; Whitmarsh et al., 2003). Characterization of topoisomerase II inhibitor-induced effects on the *MLLbcr* in lymphoblasts from a patient undergoing chemotherapy, in *ex vivo* treated peripheral blood cells from healthy individuals and *in vitro* treated leukemia cell lines, identified breakage within a telomeric *MLLbcr* fragment comprising a topoisomerase II recognition sequence (Aplan et al., 1996). Topoisomerase II inhibition by either etoposide or teniposide treatment was shown to induce the same cleavage pattern in the telomeric *MLLbcr* region in lymphoblastoid cells, T47D breast cancer cells, and human fibroblasts (Mirault et al., 2006). Causal involvement of topoisomerase II was supported by use of cells with mutated topoisomerase II showing diminished breakage (Mirault et al., 2006; Cowell et al., 2012) and by protein expression inducing hypersensitivity to etoposide (Tamaichi et al., 2013).

Despite these well-supported links between topoisomerase II action and *MLLbcr* breakage, several lines of evidence indicate that topoisomerase II is not essential for this process. First, using various human leukemia and other cancer cell lines as well as mouse embryonic fibroblasts, several groups showed that *MLLbcr* cleavage depends on apoptosis (but not necrosis) rather than topoisomerase II, even when cells are treated with topoisomerase II inhibitors (Stanulla et al., 1997; Betti et al., 2005; Hars et al., 2006). It was hypothesized that some cells can escape the cell death process and therefore transform apoptosis-induced DNA damage into leukemic translocations (Vaughan et al., 2002). In line with this, it is possible to induce *MLLbcr* breakage and rearrangements in cultured cells with a wide range of compounds not targeting topoisomerase II, such as with topoisomerase I inhibitor camptothecin, DNA polymerase inhibitor aphidicolin, microtubule inhibitor vinblastine, antimetabolites (5-fluorouracil, cytosine arabinoside, methotrexate), alkylating agents (melphalan, methylmethanesulfonate), the cross-linking agent cisplatin, with ionizing irradiation, and with various other genotoxic compounds with less defined modes of action (Stanulla et al., 1997; Ploski and Aplan, 2001; Sim and Liu, 2001; Betti et al., 2005; Le et al., 2009; Gole et al., 2014; Ireno et al., 2014; Kraft et al., 2015). It has even been reported that *MLLbcr* breakage and rearrangements can be induced by some non-genotoxic agents such as N-methylformamide (Ploski and Aplan, 2001) or by estrogen (Le et al., 2009), though for most non-genotoxic compounds this seems not to be the case (Ireno et al., 2014). Clinical data also describe 11q23/*MLL* rearrangements in patients treated with chemotherapies not addressing topoisomerase II (Faller et al., 2009; Zámečníkova, 2011). Most importantly, catalytic topoisomerase II inhibition by merbarone prior to formation of the cleavable complex or RNA interference-mediated silencing of the enzyme did not abrogate *MLLbcr* cleavage downstream of different genotoxic treatments (Betti et al., 2005). In this context it is important to note that topoisomerase II poisoning by epipodophyllotoxins like etoposide relies on the formation of a complex composed of the drug, DNA, and topoisomerase II blocking DNA re-ligation and resulting in breakage, i.e., genotoxic stress (Felix et al., 1995).

From these observations it was proposed that signaling events downstream of topoisomerase II-induced DNA double-strand break (DSB) formation are the cause of *MLLbcr* breakage and/or that several distinct enzymes can be involved (Stanulla et al., 1997; Mirault et al., 2006). The most widely discussed signaling mechanism, which underlies *MLLbcr* breakage is apoptosis leading to apoptotic DNA fragmentation (Stanulla et al., 1997; Betti et al., 2001; Sim and Liu, 2001). This two-step process of DNA degradation during apoptosis is executed by so-called apoptotic nucleases, primarily by caspase-activated DNase (CAD) but also Artemis, DNase I, DNase II, DNase γ, Endonuclease G, topoisomerase II, and cofactors such as apoptosis-inducing factor (AIF) and cyclophilins (Samejima and Earnshaw, 2005; Britton et al., 2009; Widlak and Garrard, 2009). Accordingly, *MLLbcr* cleavage could be part of early DNA fragmentation into high molecular weight (50–300 kb) fragments. Deregulated checkpoint control might permit repair of the resulting DSBs and thus survival, while erroneous DSB repair might rearrange *MLL* in some cells mediating malignant transformation (Stanulla et al., 1997; Hars et al., 2006). Indeed, genomic mapping revealed that the ~600 bp *MLLbcr* hotspot for therapy-related translocations contains not only topoisomerase II but also topoisomerase I and apoptotic cleavage sites (Mirault et al., 2006) and was shown to be DNase I-sensitive (Strissel et al., 1998). In further support of the concept of apoptotic fragmentation-driven *MLL* rearrangements, incidence of *MLL* fusions was decreased in etoposide-treated mouse embryo fibroblasts from CAD knockdown mice or after application of the pan-caspase inhibitor zVAD-fmk (Hars et al., 2006). Corresponding results were obtained in lymphoblastoid TK6 cells after apoptosis induction with anti-CD95-antibody and zVAD-fmk exposure (Betti et al., 2003; Le et al., 2009), indicating that induction of *MLL* breakage and rearrangements are observed downstream of receptor-mediated and mitochondrial pathway-dependent apoptotic signaling.

However, caspase activation seems not to be required for *MLL* breakage and rearrangements in each cellular setting. Etoposide-induced *MLLbcr* breakage was caspase-independent in CEM cells (Mirault et al., 2006). Further, irradiated MCF7 cells, which lack functional caspase-3 and thus CAD activation, show the same *MLLbcr* fragmentation as TK6 cells treated with anti-CD95 antibody (Betti et al., 2005). In our recent work, we used T47D cells, which are known to mimic the *MLLbcr* cleavage pattern of lymphoblastoid cells after genotoxic treatment (Mirault et al., 2006) and to be particularly responsive to replication stress induced by aphidicolin. Again, we found that *MLLbcr* cleavage is caspase-independent after aphidicolin treatment despite active apoptosis signaling (Gole et al., 2014). Moreover, in the context of enhanced replication stress, caspase-independent Endonuclease G rather than caspase-activated CAD, topoisomerase I, DNase I, DNase II, or Artemis was essential for *MLLbcr* rearrangements in T47D and HeLa cells (Gole et al., 2014). Replication stress is one of the outcomes of topoisomerase II-inhibitory treatments, as topoisomerase II action is required for relaxation of supercoiled DNA during replication (Felix et al., 1995). Moreover, epipodophyllotoxins generate reactive oxygen species that create various DNA lesions ultimately blocking replication (Berquist and Wilson, 2012). Therefore, erroneous responses to replication stress associated with replication fork stalling are a possible additional explanation for *MLLbcr* breakage and rearrangements after topoisomerase II inhibition using epipodophyllotoxins.

## Properties of the *MLLbcr* locus

Collective data showing that *MLLbcr* breaks and rearrangements most likely can be induced by topoisomerase II- or apoptosis/caspase-mediated but also independent mechanisms spurred the search for a common denominator, which could explain breakability of *MLLbcr* under all these different conditions. Clues might come from the intrinsic properties of the *MLLbcr* region itself. Soon after the discovery of the involvement of the *MLL* locus in leukemogenesis it was proposed that the chromatin structure of the region might play a role. Thus, the telomeric half of the *MLLbcr*, which harbors topoisomerase II-sensitive sites was described to have properties of a scaffold/matrix-attachment region (SAR/MAR) (Broeker et al., 1996). Later on, however, it was noticed that the *MLLbcr*-DNA breaks showed a higher density outside of high-affinity SAR/MAR fragments, probably because SAR/MAR-DNA is protected by a high protein content (Strissel et al., 2000; Hensel et al., 2001). Similarly, the breakpoint cluster region in the *AFF1/AF4* gene, a common translocation partner of *MLL*, also contains MAR features but again with an inverse correlation between breakage and high-affinity SAR/MAR fragments (Hensel et al., 2001). On the other hand it was discovered that the telomeric part of the *MLLbcr* has characteristics of open chromatin with high DNase I sensitivity and low histone H1 content (Strissel et al., 1998; Khobta et al., 2004). DNase I sensitivity peaks in the *MLLbcr*, similarly as in the promoter of the *MLL* gene, is positioned in the 5′-half of exon 12, just 3′ to the major therapy-related translocation breakpoint (Figure 1C). The breakpoint cluster region of *AFF1/AF4* also showed DNase I sensitivity (Strick et al., 2006), so that the therapy/infant-related *MLLbcr* breaks and rearrangements could be due to increased accessibility of this translocation hotspot for nucleases such as topoisomerase II and Endonuclease G. No such features were described for the centromeric part of the *MLLbcr* (Figure 1B), suggesting that the mechanisms of *MLL* breakage leading to *de novo* AL are quite different from the ones in t-AL and infant ALL.

At first glance contradictory to the proposed impact of the chromatin context on the fragility of the *MLLbcr*, experiments with extrachromosomal episomes containing the therapy-related *MLLbcr* fragment or with randomly chromosomally integrated extra *MLLbcr* copies showed similar cleavage and rearrangements of exogenous and the endogenous *MLLbcr in situ* (Stanulla et al., 2001; Boehden et al., 2004; Gole et al., 2014). Still, when investigating a randomly integrated copy of the therapy-related *MLLbcr* fragment, we noticed dependency of breakage and rearrangements on RNF20 (Gole et al., 2014), a histone H2B monoubiquitinase whose activity has been linked with increased chromatin accessibility (Fierz et al., 2011). This RNF20 influence was not found when analyzing extrachromosomal *MLLbcr* fragment stability (Gole et al., 2014). Further arguing against a mere *cis*-regulatory effect of the *MLLbcr* DNA sequence, we saw differences in the inducibility of rearrangements between cell clones with single copies of the *MLLbcr* integrated into different chromosomal sites in K562 myleoid leukemia cells (Ireno et al., 2014). Taken together, accessibility of naked or poorly chromatinized DNA within extrachromosomal episomes or open chromatin at intrachromosomal loci seem to significantly contribute to the t-AL/infant ALL-related *MLLbcr* breaks.

On the other hand, several pieces of evidence argue against chromatin accessibility as the sole reason for *MLLbcr* fragility, as at least two more segments of the *MLL* gene which are not recurrently rearranged have DNase I sensitive sites, namely within the promoter region of exon 1 and within exon 37 (Figure 1A). Interestingly, when murine *Mll* was introduced into human cells on an episome, it was cleaved at the site of the endogenous *Mll* in murine cells, which was the first hint that the sequence of *MLL* itself plays an important role in breakage distribution (Stanulla et al., 2001). Later on it was discovered that *MLLbcr* breaks at non-random positions at a sequence reminiscent of nick-forming sequences, i.e., hypersensitive regions positioned regularly at loop-size intervals in the eukaryotic chromatin, which are targeted during high-order apoptotic DNA fragmentation (Székvölgyi et al., 2006). These sequences may form secondary structures such as hairpins and these hairpins were proposed to represent the basis of *MLLbcr* fragility (Székvölgyi et al., 2006). Indeed, a hairpin secondary structure was described at a topoisomerase II cleavage site at the 5′-terminus of *MLL* exon 12, with positioning of the topoisomerase II site in the loop and the actual breakpoint at the stem of the hairpin (Le et al., 2009). A broader look at the intron 11/exon 12 boundary reveals that the whole region harboring major therapy-related translocation breakpoints is predicted to fold into a complex secondary structure with hairpins (Figure 1D). Such non-B DNA structures can form during DNA replication when longer runs of single-stranded DNA are transiently formed as well as in a replication-independent manner (Wang et al., 2013). When left unresolved, such structures can obstruct progression of the DNA replication and/or RNA transcription machinery (Le et al., 2009; Wang et al., 2013), both of which could contribute to *MLLbcr* breakage and further rearrangements (Le et al., 2009) as discussed in more in detail below.

## Replication stalling or transcription stalling?

### Replication stalling

Breakage of the therapy-related *MLLbcr* hotspot in response to treatment with aphidicolin, an inhibitor of polymerase α, δ, and ε (Ozeri-Galai et al., 2011), indicated involvement of replication stress in *MLLbcr* destabilization, particularly as apoptosis induction was not necessarily coupled with treatment (Gole et al., 2014). Together with formation of a stable secondary structure predicted to halt the progression of replication forks (Figure 1D), these *MLLbcr* features were reminiscent of so-called fragile sites (Durkin and Glover, 2007; Ozeri-Galai et al., 2011; Debatisse et al., 2012). Fragile sites by definition are loci with frequent breaks under replication stress. While Rare Fragile Sites (RFS) are found only in few individuals such as with micro- or mini-satellite repeat expansion-associated inheritable diseases, Common fragile sites (CFS) are population-wide hotspots for chromosomal rearrangements, whereby >200 were mapped in humans (Letessier et al., 2011; Debatisse et al., 2012). CFS were discovered as gaps and constrictions in metaphase chromosomes of cells grown under mild replication stress conditions following treatment of cells with aphidicolin (Glover et al., 1984). Further work showed that CFS are also responsive to replication impediments such as due to secondary structures, decelerated replication such as due to depletion of nucleotide pools, perturbations of replication due to failure of origin firing or deregulated checkpoint control mechanisms, and interference with transcription (Letessier et al., 2011; Ozeri-Galai et al., 2011; Debatisse et al., 2012). Genomic analysis revealed that CFS are prone to sister chromatid exchange, loss of heterozygosity, deletions, and tandem segmental genomic duplications. Intriguingly, CFS correlate with chromosomal breakpoints in tumors. Altogether, even though not formally defined as CFS, the therapy-related *MLLbcr* hotspot shares crucial characteristics with these fragile sites.

Depending on the cause, severity, and persistence of DNA replication stress it emerges as stalling and/or collapse of DNA replication forks. DNA lesions represent an obstacle to continued fork progression and therefore are a major source of replication stress. Replication stalling can directly create DSBs, when the fork encounters a single-stranded DNA break. However, DSBs may also arise during replication reactivation, when damage bypass mechanisms transiently introduce breaks such as during inter-strand DNA cross-link repair or when strand exchange intermediates are created that are vulnerable to incisions by structure specific nucleases such as upon replication fork reversal (Atkinson and McGlynn, 2009; Thompson and Hinz, 2009). Endogenous and exogenous reactive oxygen species are a prominent source of a wide spectrum of lesions ranging from base modifications to inter-strand DNA cross-links (Berquist and Wilson, 2012; Ensminger et al., 2014). Other replication blocking insults may arise from environmental, nutritional, and life-style risk factors, as well as from therapeutic interventions such as chemo- and radiotherapies, i.e., the well-described cause of secondary leukemia associated with *MLLbcr* rearrangements (Shivakumar et al., 2008; Pullarkat et al., 2009; Shim et al., 2010; Abdulwahab et al., 2012; Cowell and Austin, 2012). The combination of deregulated replication and genotoxic stress such as in case of oncogene- or hormone-induced proliferation together with reactive oxygen species and reactive metabolite formation, respectively, seems to be particularly detrimental to the integrity of the genome (Bolton and Thatcher, 2008; Macheret and Halazonetis, 2015).

Replication stress-induced rearrangements could also be the common trigger for rearrangements within the telomeric *MLL* fragment causing both therapy-related and infant ALL (Broeker et al., 1996; Cimino et al., 1997; Reichel et al., 2001; Cowell and Austin, 2012; Meyer et al., 2013). HSCs and progenitor cells, i.e., the cells-of-leukemia-origin, are known to exit from quiescence into an active cycle upon infection, enforced self-renewal due to bone marrow transplantation, and in response to genotoxic insults. A recent publication provided *in vivo* evidence in mice for the appearance of DSBs and single-stranded breaks as a direct consequence of HSC cell cycle entry under conditions mimicking viral infection (Walter et al., 2015). Serial transplantation of HSCs into immunodeficient mice as well as clinical HSC transplantation were shown to trigger replication stress and persistent DNA damage (Yahata et al., 2011). The authors identified mitochondrial reactive oxygen species as mediator of replication stress-induced DNA damage in both cases. Proliferative stress further correlated with genomic instability in bone marrow transplants during repopulation of recipients favoring secondary tumor formation (Hertenstein et al., 2005; Flynn and Kaufman, 2007). Murine HSCs, when pushed out of quiescence to reconstitute mature blood cells by massive self-renewal following irradiation or chemotherapeutic treatment show hypersensitivity to further genotoxic treatment and proneness to lymphomagenesis (Cheshier et al., 1999; Labi et al., 2010; Trumpp et al., 2010; Desai et al., 2014). *MLLbcr* rearrangements in t-AL and infant ALL associated with radio-/chemotherapy and fetal hematopoiesis, respectively, are thus compatible with HSC exit from quiescence and hypersensitivity to replication-associated damage. Further clues to an involvement of replication stress in *MLLbcr* rearrangements came from Fanconi anemia (FA) patients, as the FA pathway is central to the stabilization and reactivation of replication forks (Thompson and Hinz, 2009). The integrity of the FA pathway was reported essential to prevent high rates of HSC death upon replication stress (Ceccaldi et al., 2012; Walter et al., 2015). Supporting a causal role of replication-associated damage in *MLLbcr* rearrangements, therapy-related *t*_(11; 16)_-AML with a *MLL*-*CBP* fusion was observed in a pre-B-cell ALL pediatric patient with FA (Sugita et al., 2000), and *MLL* partial tandem duplications (*MLL-PTD*) were reported in bone marrow samples from FA patients (Quentin et al., 2011).

### Transcription stalling

As with replication stalling, various hurdles ranging from repetitive sequences to chromatin changes may also cause transcription stalling and several pieces of evidence suggested involvement of transcription stalling in *MLLbcr* destabilization. Using chromatin immunoprecipitation (ChIP) in Jurkat immortalized human T-lymphocytes and human CD34^+^ HSPCs, the telomeric *MLLbcr* region encompassing the therapy-related hotspot at intron 11/exon 12 was characterized by chromatin marks reminiscent of promoters with the lowest histone H1 content and H4-acetylated islands (Khobta et al., 2004). In 2007, Marschalek and co-workers (Scharf et al., 2007) confirmed a chromatin structure that supports active transcription. Even further, they proposed that this telomeric *MLLbcr* fragment colocalizes with a gene-internal promoter resulting in a truncated *MLL* protein. Interestingly, the authors detected a similar putative gene-internal promoter in the homologous region in the murine *Mll*. Intriguingly, etoposide-induced break sites were then mapped at the RNA polymerase II binding and transcription initiation site within the putative gene-internal promoter in Jurkat and REH human cell lines as well as peripheral blood mononuclear cells (Scharf et al., 2007). However, no such information on a gene-internal promoter at intron 11/exon 12 is included in current Ensembl data (release 79, March 2015, Cunningham et al., 2015) which assembles information from 18 different human cell lines (including K562, DND-41 leukemic T-cells and GM12878 B-lymphocytes). Nevertheless, a DNase I sensitive open chromatin structure was detectable in the critical region. It further coincides with binding sites for CTCF that can function as a transcription factor, demarcate chromatin domains, and mediate chromosomal looping interactions (Figures 1B,C). One possibility to reconcile the different observations could be cell type-dependent regulatory effects. Importantly, replication and transcription cannot be looked upon separately, because collisions between transcription and replication machineries or RNA-DNA hybrid (R-loops) formed during transcription are known to impede replication fork progression (Macheret and Halazonetis, 2015). Conflicts between transcription and replication can stem from deregulated checkpoint control mechanisms changing the length of cell cycle phases and the program of origin firing during malignant transformation processes.

Different views exist also regarding the order of events leading to DNA breakage and subsequent rearrangements at transcriptionally active sites. The two major hypotheses imply either that rearrangements occur after DNA damage at the juxtaposed sequences/genes (contact first) or due to increased mobility of distant sequences/genes after DNA damage (breakage first). The contact first hypothesis implies transcription stalling as the basis of DNA break formation. In support of such a model regarding *MLL* rearrangements, it was noticed that *MLL* transcripts can be founded in close proximity to actively transcribed translocation partners *AF4* and *AF9* in KG1 myeloid leukemia cells and Nalm-6 pre-B leukemia cells and *AF4* and *AF9* transcripts in close proximity to actively transcribed *MLL* (Cowell et al., 2012). This suggests that these genes could be part of common transcription factories, where simultaneous DNA breaks on translocation partners in close proximity are possible. Supporting the common transcription factory/contact first hypothesis are also observations of *MLL*-*AF4* mRNA fusion transcripts in the absence of the corresponding gene fusion in both tumor and normal hematopoietic cells from infant ALL patients (Uckun et al., 1998) and in peripheral blood mononuclear cells from healthy individuals (Kowarz et al., 2011). These observations were interpreted such that premature termination of transcription may trigger intra- and to a lesser extent inter-genic trans-splicing events resulting in fusion transcripts at common transcription factories. Due to the intriguing coincidence of trans-splicing and translocation events involving the same gene pairs, and inspired by findings from yeast on RNA-templated DNA repair (Storici et al., 2007), the authors proposed that fusion transcripts may in fact guide error-prone repair of DNA lesions arising in one of the translocating genes (Kowarz et al., 2011, 2012). In a recent study addressing the role of prenatal hormone exposure in *MLL* translocations leading to infant ALL, estradiol-induced *MLL* and *MLLT3/AF9* colocalization as well as fusion transcript formation were detected. Interestingly, this process required the protein activation-induced cytidine deaminase, AID (Wright et al., 2014). Even though primarily known to be involved in targeted *Ig* gene maturation processes, AID has also been shown to exert unspecific genotoxic effects upon activation by estrogen (Pauklin et al., 2009). ChIP experiments revealed localization of AID in *MLLbcr* intron 11 (Wright et al., 2014), to where it may become recruited by stalled RNA polymerase in analogy to the situation during *Ig* gene switching (Pavri et al., 2010). Given that aberrantly activated AID can cleave at 150 target sites outside of *Ig* genes including well-described translocation hotspots (Hakim et al., 2012), it represents a key candidate for involvement in genome rearrangements following transcription stalling.

The breakage first hypothesis positions contact formation as an event secondary to DNA break formation. One of the reasons arguing against the contact first hypothesis was that 3D-FISH analysis indicated closer spatial proximity of *MLL* and *ENL* genes in interphase nuclei of myeloid (AML-193, PLB-985) and lymphoid cells (Nalm-6, IM-9) as compared with *MLL* and *AF4* genes, even though *AF4* represents the most frequent partner in *MLL* translocations (Gué et al., 2006). Evidence supporting breakage as the primary event leading to chromosome rearrangements was obtained also with the 3D time-lapse microscopy in living U2OS osteosarcoma cells using the fluorescently labeled 53BP1-GFP protein as a DSB marker. These experiments revealed enhanced mobility of damaged as compared with intact chromatin domains and susceptibility of only DSB containing chromatin to retardation of the movements by agents that affect chromatin organization (Krawczyk et al., 2012). Glukhov and colleagues (Glukhov et al., 2013) more specifically addressed the mobility of the *MLLbcr* in etoposide-treated Jurkat cells. The authors observed that the telomeric *MLLbcr* DNA end moved out of the chromosome 11 territory within 1 h following cleavage, which could increase the probability to meet and erroneously merge with a translocation partner.

**Figure 2:**
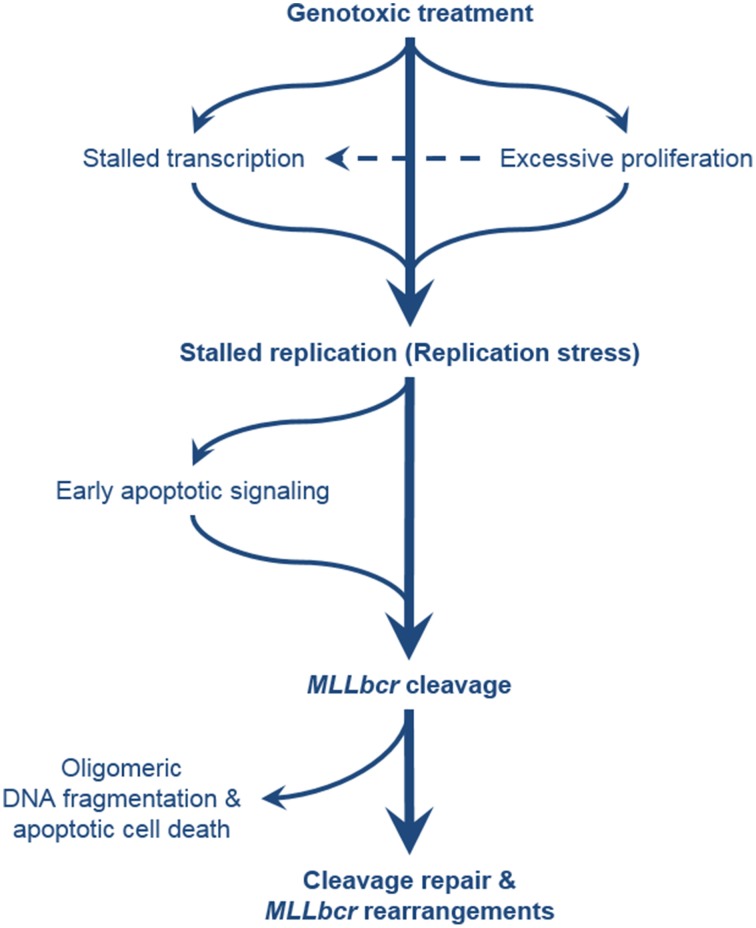
**Comprehensive overview of the pathways leading to** ***MLLbcr*** **rearrangements**. Exposure of the cells to genotoxic agents can directly or indirectly cause stalling of DNA replication, e.g., by antimetabolite treatment, and by formation of DNA adducts and downstream repair intermediates (e.g., by treatment with alkylating agents and downstream excision repair) or transcription stalling, respectively. Excessive proliferation of the hematopoietic stem/progenitor cells (cells-of-leukemia-origin) due to enforced self-renewal after bone marrow transplantation or genotoxic insults also results in replication stress. *MLLbcr* in turn is cleaved either as part of the attempt to rescue stalled forks or as part of DNA damage-induced early apoptotic high order DNA fragmentation. DSBs at the *MLLbcr* can be repaired through NHEJ, MMEJ, or homology-directed repair which can lead to leukemogenic rearrangements preventing further oligomeric DNA fragmentation and cell death.

Observations made on the frequency of *Igh* translocations in murine B lymphocytes may reconcile the two models in that they showed that site-directed DNA break formation is strictly associated with translocations, whereas in the absence of targeted DNA breaks rearrangements are related to the contact frequency with the partner genes, i.e., reflect the nuclear architecture (Hakim et al., 2012). Thus, targeted cleavage seems to govern downstream events. Notably, *MLLbcr* cleavage and at least intramolecular rearrangements do not require higher-order chromatin structures, take place on non-replicating episomes and independently of the *MLLbcr* sequence orientation with respect to a neighboring transcriptional promoter arguing against an essential role of replication or transcription stalling immediately within the *MLLbcr* (Stanulla et al., 2001; Boehden et al., 2004; Gole et al., 2014). However, chromosomal structures and processes promote *MLLbcr* breakage in *cis* and/or influence cleavage in *trans* such as via early apoptotic signaling and activation of Endonuclease G (Gole et al., 2014) (Figure 2). Intriguingly, damaged DNA and complex DNA structures, such as R-loops, all of which can promote replication and/or transcription stalling are preferential targets of Endonuclease G-mediated cleavage (Ruiz-Carrillo and Renaud, 1987; Ohsato et al., 2002; Kalinowska et al., 2005) making it a strong candidate for the nuclease responsible for *MLLbcr* cleavage.

## How is broken *MLLbcr* repaired?

The striking disparity in the distribution of breakpoints associated with *de novo* vs. therapy-related AL clearly involves differential targeting of DNA breaks but may additionally be influenced by the involvement of specific DNA repair mechanisms (Broeker et al., 1996). So far, most of the information on the involvement of DNA repair pathways originated from the mapping of fusion points and the analysis of rearranged sequences. In this way, Alu repeat-mediated homology-directed repair was shown to create partial tandem *MLL* duplications in a group of *de novo* AML patients (So et al., 1997; Strout et al., 1998). The corresponding Alu elements are present in *MLL* intron 1 and in the centromeric part of the *MLLbcr*, and consequently are not involved in therapy-associated rearrangements mostly affecting the telomeric part of the *MLLbcr*. On the other hand, collective data from several groups were interpreted such that non-homologous end-joining (NHEJ) is the major mechanism for the creation of translocations. Sequence analysis of intergenic fusions frequently lacked overlapping homologies or revealed micro-homologies (mini-direct repeats) rather than extended homologies, which pointed to misrepair by error-prone classical NHEJ or alternative microhomology-mediated end joining (MMEJ) mechanisms (Reichel et al., 2001; Whitmarsh et al., 2003; Le et al., 2009). These features were detected in infant and adult ALL as well as t-AL patients and consequently in centromeric and telomeric *MLLbcr* regions. More specifically, *MLL*-*AF4* fusions in 40 ALL patients diagnosed with *t*_(4;11)_ showed filler DNA of ≤21 nucleotides and mini-direct repeats of ≤7 nucleotides at the inter-chromosomal junctions in more than half of the cases. Additionally, duplications of ≤463 nucleotides of parental sequences, inversions of ≤267 nucleotides, and/or deletions of ≤4413 nucleotides were found (Reichel et al., 2001). Analysis of a t-AML case diagnosed with *t*_(9;11)_ revealed a *MLL*-*AF9* translocation fusing a micro-homologous TATTA sequence without gain or loss of any further nucleotides (Whitmarsh et al., 2003). Analysis of blood samples from breast cancer and lymphoma patients undergoing chemotherapeutic treatments identified *MLL* rearrangements with micro-homologous sequences of 2–8 bp in most of the junction sequences (Le et al., 2009). Even *ex vivo* flavonoid exposure of primary human CD34^+^ HSPCs was demonstrated to induce DSBs and translocations in the *MLL* intron 11/exon 12 region mostly via error-prone repair involving micro-homologies but in fewer cases via homology-directed repair at Alu-like sequences (van Waalwijk van Doorn-Khosrovani et al., 2007). Interestingly, in almost all presumed MMEJ cases repetitive LINE/L1 or SINE/Alu-like elements were detected adjacent to the breakpoints on at least one side and short palindromic sequences predicted to be formed. When Zinc Finger Nuclease technology was used to introduce cuts precisely within the *MLL*, detrimental genetic changes were only observed upon additional inhibition of the key NHEJ factor DNA-PK, which was accompanied by a rise in homology-directed activities (Do et al., 2012). This result suggested that clean breaks within the *MLLbcr* less frequently cause leukemogenic rearrangements, because they are quickly removed by classical NHEJ. Biochemical analysis indeed demonstrated association of DNA-PK with the *MLLbcr* following ionizing irradiation-induced breakage (Betti et al., 2001).

With the advance of technologies—starting from the analysis of cytogenetically defined karyotypes toward long-distance inverse PCR-based sequence analysis—detection of genomic changes has seen a dramatic sensitivity rise. Reflecting higher resolution, the spectrum of newly described *MLLbcr* rearrangements became larger in recent years. Previously, *MLL* was known to predominantly participate in reciprocal chromosomal translocations. More recently, non-reciprocal rearrangements resulting in insertions, inversions, and deletions were identified to account for a significant fraction of *MLLbcr* rearrangements. Complex rearrangements requiring at least tree DSBs and involving at least two chromosomes were observed to account for a fraction of up to 26% of *MLL* rearrangements. Complex rearrangements may result in three-way translocations, chromosomal translocations associated with deletions or with fragment insertions. In the most extreme case of complex chromosomal translocations many fusion alleles are generated during a single cell division in a process called chromothripsis (De Braekeleer et al., 2011; Cerveira et al., 2012; Meyer et al., 2013).

A major source of non-reciprocal complex rearrangements is break-induced replication, whereby replication fork collapse can be induced by ionizing irradiation or oncogenes followed by processing into one-ended DSBs, which are repaired by break-induced replication, a form of homology-directed repair, which involves replication template switching (Macheret and Halazonetis, 2015). Desai et al. (2014) demonstrated reliance of HSPCs, the cells-of-leukemia-origin, on homology-directed repair for DSB processing upon exit from quiescence. Thus, pushing HSPCs of homology-directed repair-deficient *Exo1* mutant mice into the cell cycle by treatment with 5-Fluorouracil or poly-IC caused hypersensitivity to ionizing radiation. There is also evidence for *MLLbcr* rearrangements by the homology-directed repair mechanism homologous recombination, i.e., the DSB repair pathway considered to be most error-free. First, sequence analysis of partial tandem duplications of the *MLL* gene in AML patients suggested recombination between imperfectly homologous Alu sequences through generation of a heteroduplex fusion (Strout et al., 1998). Second, knockdown experiments of replication stress-induced *MLLbcr* rearrangements indicated involvement of RAD51 (Gole et al., 2014). Third, a pathogenic role of altered ATM function was proposed, when a germline missense *ATM* mutation was detected in the phosphatidylinositol-3 (PI-3) kinase coding region of a pediatric leukemia patient with *MLL* rearrangement (Oguchi et al., 2003). ATM deficiency was further shown to result in excessive binding of the DNA recombination protein RAD51 at the *MLLbcr* translocation breakpoint hotspot of 11q23 chromosome translocation after etoposide exposure. Binding of Replication protein A (RPA) and the chromatin remodeler INO80, which facilitate RAD51 loading on damaged DNA, to the hotspot were also increased by ATM deficiency. Thus, in addition to activating DNA damage signaling, ATM may avert chromosome translocations by preventing excessive loading of recombinational repair proteins onto translocation breakpoint hotspots (Sun et al., 2010). In this context it is of interest that reduced ATM levels were found in cycling human HSPCs as compared with cycling mature human peripheral blood lymphocytes (Kraft et al., 2015), providing one possible explanation for HSPC proneness to aberrant homologous recombination events at the *MLLbcr*.

## Conclusions

Therapy-induced leukemia represents the most severe side effect of chemotherapy after successful treatment of the primary tumor. Evaluation of population-based data for the years 1975–2008 from nine US cancer registries revealed a t-AML incidence of 5.7% among the 29% of cancer patients who had received initial chemotherapy (Morton et al., 2013). During the observation period the t-AML risk rose among some cancer patients like non-Hodgkin lymphoma (NHL) patients, while it declined among others like ovarian carcinoma patients. Changes were explained by more frequent administration of subsequent therapies for persistent or relapsed NHL and a shift from an alkylating (melphalan) to a crosslinking (platinum) drug-based chemotherapy of ovarian carcinoma, documenting the influence of cumulative dose and the mode-of-action of the drug. Accordingly, attempts to optimize treatment modalities have focused on dose- and drug-adaptation in analogy to reduced-intensity conditioning regimens for patients requiring bone marrow transplantation (Myers and Davies, 2009). However, a recent study showed that the risk of secondary malignancies did not decline with reduced-intensity and reduced-toxicity conditioning regimens (Shimoni et al., 2013). Moreover, the benefits from modern medicine with a 3% annual increase of successfully treated cancer survivors, call our attention to the predicted rise in secondary malignancy risk (Maddams et al., 2011; Dong and Chen, 2014). Knowing that age is the most prominent cancer risk factor, the worldwide rise in longevity will further increase cancer incidences. Additionally, the hematopoietic system accumulates replication stress damage with age (Flach et al., 2014), which may rise the vulnerability for chemotherapy-induced *MLLbcr* rearrangements (Gole et al., 2014). So far, the benefits of chemotherapy significantly outweigh the risk of adverse effects (Dong and Chen, 2014). Novel targeted approaches such as PARP or mTOR inhibitor therapies inducing replication stress and EndoG nuclear translocation, respectively, may also bear a risk of *MLLbcr* destabilization (Gole et al., 2014). Hopes for better survival of patients with *MLL*-rearranged leukemia come from novel treatment regimens such as a triple immunotherapy targeting tumor-associated antigen and natural killer cell resistance (Chan et al., 2012). The challenge for the future will, however, be to identify markers for secondary leukemia risk and to develop compounds preventing t-AML for rational design of personalized combination therapies.

### Conflict of interest statement

The authors declare that the research was conducted in the absence of any commercial or financial relationships that could be construed as a potential conflict of interest.
